# Rubiadin Mediates the Upregulation of Hepatic Hepcidin and Alleviates Iron Overload via BMP6/SMAD1/5/9-Signaling Pathway

**DOI:** 10.3390/ijms26031385

**Published:** 2025-02-06

**Authors:** Xueting Xie, Linyue Chang, Xinyue Zhu, Fengbei Gong, Linlin Che, Rujun Zhang, Lixin Wang, Chenyuan Gong, Cheng Fang, Chao Yao, Dan Hu, Weimin Zhao, Yufu Zhou, Shiguo Zhu

**Affiliations:** 1 Department of Immunology and Pathogenic Biology, School of Integrative Medicine, Shanghai University of Traditional Chinese Medicine, Shanghai 201203, China; xiexueting222@163.com (X.X.); suzyzhuxy@163.com (X.Z.); 13324366877@163.com (L.C.); wanglixin@shutcm.edu.cn (L.W.); gongchenyuan0623@aliyun.com (C.G.); 15755181380@163.com (C.F.); yaochao87@hotmail.com (C.Y.); 2 Center for Traditional Chinese Medicine and Immunology Research, School of Integrative Medicine, Shanghai University of Traditional Chinese Medicine, Shanghai 201203, China; 3 Natural Product Research Center, Shanghai Institute of Materia Medica, Chinese Academy of Sciences, Shanghai 201203, China; changlinyue@simm.ac.cn (L.C.); gongfengbei@simm.ac.cn (F.G.); rujunzhang@simm.ac.cn (R.Z.); wmzhao@simm.ac.cn (W.Z.); 4 University of Chinese Academy of Sciences, No. 19A Yuquan Road, Beijing 100049, China; 5 School of Acupuncture, Moxibustion and Tuina, Shanghai University of Traditional Chinese Medicine, 1200 CaiLun Rd., Shanghai 201203, China; hudanrose@shutcm.edu.cn

**Keywords:** rubiadin, hepcidin, iron metabolism, iron overload, BMP6/SMAD1/5/9-signaling pathway

## Abstract

Iron overload disease is characterized by the excessive accumulation of iron in the body. To better alleviate iron overload, there is an urgent need for safe and effective small molecule compounds. Rubiadin, the active ingredient derived from the Chinese herb Prismatomeris tetrandra, possesses notable anti-inflammatory and hepatoprotective properties. Nevertheless, its impact on iron metabolism remains largely unexplored. To determine the role of rubiadin on iron metabolism, Western blot analysis, real-time PCR analysis, and the measurement of serum iron were performed. Herein, we discovered that rubiadin significantly downregulated the expression of transferrin receptor 1, ferroportin 1, and ferritin light chain in ferric-ammonium-citrate-treated or -untreated HepG2 cells. Moreover, intraperitoneal administration of rubiadin remarkably decreased serum iron and duodenal iron content and upregulated expression of hepcidin mRNA in the livers of high-iron-fed mice. Mechanistically, bone morphogenetic protein 6 (BMP6) inhibitor LDN-193189 completely reversed the hepcidin upregulation and suppressor of mother against decapentaplegic 1/5/9 (SMAD1/5/9) phosphorylation induced by rubiadin. These results suggested that rubiadin increased hepcidin expression through the BMP6/SMAD1/5/9-signaling pathway. Collectively, our findings uncover a crucial mechanism through which rubiadin modulates iron metabolism and highlight it as a potential natural compound for alleviating iron-overload-related diseases.

## 1. Introduction

Iron is essential for basic metabolic processes in cells and organisms. The oxidation state of iron in two stable cycles between the Fe^2+^ and Fe^3+^ participates in the biochemical processes within the biology [1]. For most organisms, iron is found in red blood cells, and macrophages engulf senescent red blood cells and release iron into the circulatory system [2]. This iron cycle provides most of the daily iron requirements for humans (about 20–25 mg) [1]. Typically, only 1–2 mg of iron per day is absorbed to compensate for the loss of iron [3], such as shedding of intestinal epithelial cells, skin and urine cells, blood loss, or sweating. Iron absorption can be enhanced when demand is high (for example, due to increased erythropoiesis or pregnancy) and inhibited when iron overload occurs. Iron deficiency can result in anemia, whereas iron overload leads to the accumulation of reactive oxygen species (ROS), which can cause extensive damage to multiple organs [1]. Therefore, maintaining the body’s iron homeostasis is crucial.

Hepcidin is a 25-amino acid peptide hormone secreted by hepatocytes that regulates iron metabolism throughout the body [4]. Ferroportin 1 (Fpn1) is the unique, known iron-exporting protein in mammals and is widely distributed in duodenum, liver, and spleen macrophages [5]. Hepcidin is directly bound to Fpn1 [6], leading to the ubiquitination of Fpn1 [7,8]. Therefore, the hepcidin–Fpn1 axis plays an indispensable role in the regulation of iron metabolism. Bone morphogenetic protein 6 (BMP6)/suppressor of mother against decapentaplegic 1/5/9 (SMAD1/5/9) [9] and interleukin 6 (IL-6)-induced janus kinase 2 (JAK2)/signal transducer and activator of transcription 3 (STAT3) [10,11] are the classical pathways that regulate the expression of hepcidin. Hemochromatosis is defined as hereditary systemic iron overload caused by hepcidin deficiency [12]. The main feature of the disease is normal erythropoiesis, but excessive iron accumulation in the cells of parenchymal organs, such as liver and heart, lead to organ damage. Therefore, up-regulation of hepcidin to inhibit iron absorption is a promising treatment for iron overload disease.

Traditional Chinese medicine is an important resource for new drug discovery. Motherland medical records that Prismatomeris tetrandra has the effect of cooling blood and stopping bleeding, diluting dampness and yellowing, dispersing stasis, and strengthening tendons and was used for bleeding gums, anemia, hepatitis, rheumatoid arthritis, bruises, and urinary tract infections. Rubiadin (1, 3-dihydroxy-2-methylanthraquinone), the active ingredient of the Chinese herb Prismatomeris tetrandra, has demonstrated anti-cancer, anti-inflammatory, and hepatoprotective effects [13,14]. Nevertheless, its impact on iron metabolism remains largely unexplored.

In this study, we discovered that rubiadin significantly up-regulated the expression of hepcidin and promoted the phosphorylation of SMAD1/5/9 in HepG2 cells. Additionally, we found that rubiadin significantly decreased the serum iron (SI), transferrin saturation (TF%) levels and duodenal iron content and relieved splenomegaly and increased the hepatic hepcidin in high-iron-fed mice. Finally, we confirmed that rubiadin increased the hepcidin expression through the BMP6/SMAD1/5/9-signaling pathway. Taken together, our findings proposed for the first time that rubiadin plays a crucial role in regulating iron metabolism and provided a candidate natural compound for alleviating iron overload disease.

## 2. Results

### 2.1. Rubiadin Significantly Upregulates Hepcidin Expression in HepG2 Cells

**Figure 1:**
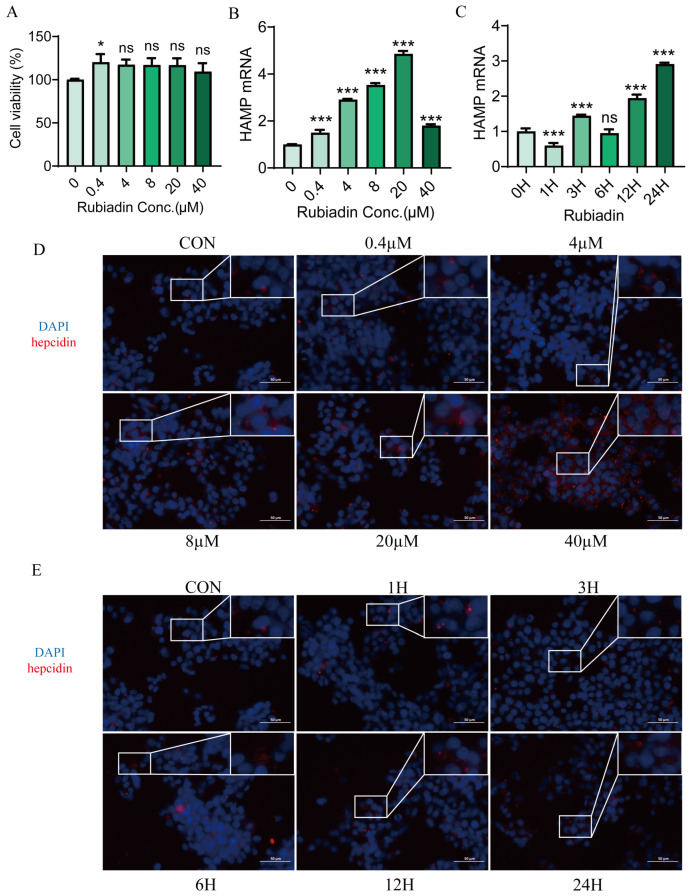
Rubiadin significantly upregulates hepcidin expression in HepG2 cells. (**A**) Effect of varying concentrations (0 to 40 μM) of rubiadin on the proliferation of HepG2 cells for 24 h. (**B**) Hepcidin expression was quantified in HepG2 cells that had been treated with the specified concentrations of rubiadin for 24 h. (**C**) The expression of hepcidin was quantified after treatment of HepG2 cells with 20 μM rubiadin at specific time points. (**D**) Hepcidin protein expression in HepG2 cells treated with the indicated concentrations of rubiadin for 24 h was evaluated using an immunofluorescence assay. (**E**) Hepcidin protein expression in HepG2 cells treated for 20 μM rubiadin for the indicated time points was evaluated via the immunofluorescence assay. Scale bars are 50 μm. Significance was calculated using a one-way ANOVA. ns, no significance, * *p* < 0.05, *** *p* < 0.001.

We treated HepG2 cells with varying concentrations of rubiadin (0–40 μM) for 24 h. The CCK8 assay demonstrated that even at the highest concentration, rubiadin did not affect the cell viability of HepG2 cells (Figure 1A). We then found that rubiadin increased hepcidin expression in a dose-dependent, with the highest being 5 times more than the control group (Figure 1B), and in a time-dependent (Figure 1C). Cellular immunofluorescence also showed the same results (Figure 1D,E). Overall, these results indicate that rubiadin upregulates hepcidin expression in a concentration- and time-dependent manner.

### 2.2. Rubiadin Decreases the Protein Expression of TfR1, Fpn1 and FtL in FAC Treated or Untreated HepG2 Cells

**Figure 2:**
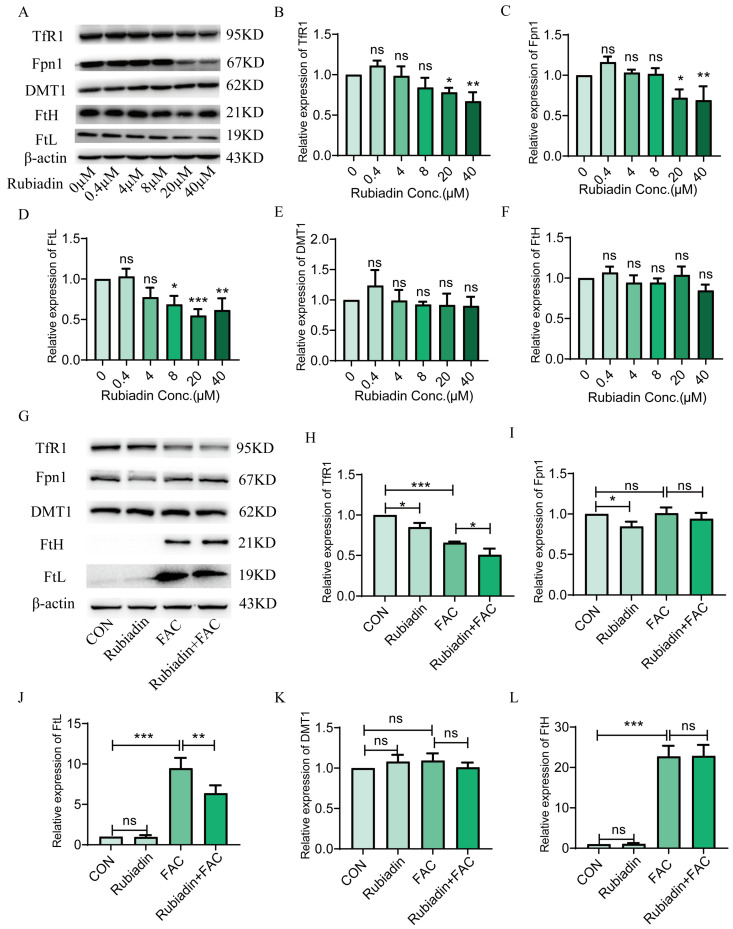
Rubiadin decreases the protein expression of TfR1, Fpn1 and FtL in FAC treated or untreated HepG2 cells. (**A**) Western blot analysis of proteins related to iron metabolism in HepG2 cells after rubiadin treatment for 24 h. Quantification of transferrin receptor 1 (TfR1) (**B**), ferroportin 1 (Fpn1) (**C**), ferritin light chain (FtL) (**D**), divalent metal transporter 1 (DMT1) (**E**), and ferritin heavy chain (FtH) (**F**) were shown. (**G**) HepG2 cells were loaded with iron by incubation with ferric ammonium citrate (FAC) (100 μM) for 24 h. After removing the medium, cells were washed and then treated with 20 μM rubiadin for 24 h. Western blot analysis of TfR1, DMT1, Fpn1, FtH, and FtL expression. Quantification of TfR1 (**H**), Fpn1 (**I**), FtL (**J**), DMT1 (**K**), and FtH (**L**) were shown. Significance was calculated using a one-way ANOVA. ns, no significance, * *p* < 0.05, ** *p* < 0.01, *** *p* < 0.001.

Next, we investigated the impact of rubiadin on proteins involved in iron metabolism. The Western blot analysis revealed that rubiadin dose-dependently decreased the protein expression of TfR1, Fpn1, and FtL (Figure 2A–D) while showing no significant effect on DMT1 and FtH (Figure 2E,F). Further research found that rubiadin reversed the elevation of FtL caused by FAC (Figure 2J), further down-regulation of TfR1 protein expression based on FAC (Figure 2H), with no effect on Fpn1, DMT1, and FtH under FAC-treated HepG2 cells (Figure 2I,K,L). Collectively, these results demonstrated that rubiadin could reduce the amount of intracellular iron, in other words, the reduction in intracellular intake is higher than that in iron efflux.

### 2.3. Rubiadin Enhances the Phosphorylation of STAT3 and SMAD1/5/9 in a Dose-Dependent Manner

**Figure 3:**
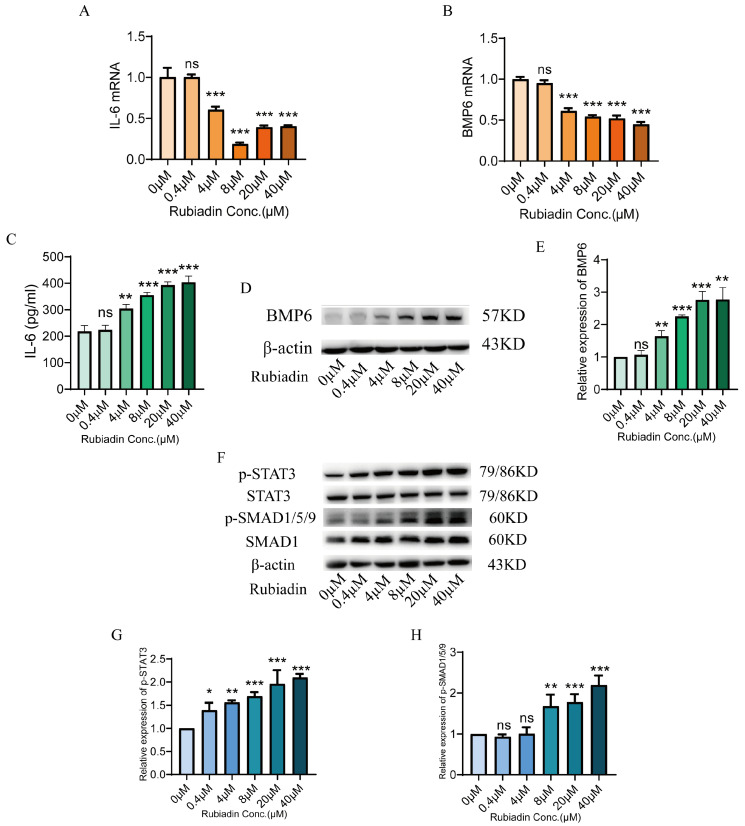
Rubiadin increased phosphorylation of signal transducer and activator of transcription 3 (STAT3) and suppressor mothers against decapentaplegic 1/5/9 (SMAD1/5/9) in HepG2 cells (**A**,**B**) Interleukin 6 (IL-6) and bone morphogenetic protein 6 (BMP6) mRNA expression were measured in HepG2 cells that were treated with the specified concentrations of rubiadin for 24 h. (**C**–**E**) IL-6 and BMP6 protein expression were measured in HepG2 cells that were treated with the specified concentrations of rubiadin for 24 h. (**F**) Phosphorylation of STAT3 and SMAD1/5/9 in HepG2 cells analyzed by Western blot after rubiadin treatment for 24 h. (**G**,**H**) Quantification of p-STAT3 (**G**) and p-SMAD1/5/9 (**H**) were shown. Significance was calculated using a one-way ANOVA. ns, no significance, * *p* < 0.05, ** *p* < 0.01, *** *p* < 0.001.

We have previously confirmed that rubiadin significantly increases the expression of hepcidin mRNA. In order to elucidate the mechanism by which rubiadin increased hepcidin expression, we examined two classical pathways, IL-6/STAT3 and BMP6/SMAD1/5/9, that regulate hepcidin. We found that IL-6 and BMP6 mRNA (Figure 3A,B) levels were significantly down-regulated by rubiadin for 24 h. However, rubiadin could increase the proteins of IL-6 and BMP6 (Figure 3C–E). Moreover, rubiadin enhanced STAT3 and SMAD1/5/9 phosphorylation in a dose-dependent manner (Figure 3F–H). Altogether, these findings suggest that rubiadin could enhance the phosphorylation of STAT3 and SMAD1/5/9.

### 2.4. Rubiadin Remarkably Reverses the Abnormal Elevation of Serum Iron and Alleviates Splenomegaly Caused by Iron Overload

In order to confirm whether rubiadin had the same in vivo effect as in vitro, first, we selected 8-week-old C57BL/6 normal mice and administered them intraperitoneal injections of rubiadin at a dose of 1 mg/kg every other day for 6 weeks. Our results showed that rubiadin significantly reduced SI, TIBC, and TF% in normal mice (Figure S1). This prompted us to investigate whether rubiadin has a similar effect on iron overload mice.

We then used a high-iron diet to induce iron overload condition in C57BL/6 mice. Six-week-old normal mice were treated a high-iron diet for two weeks. Compared to mice fed a normal diet, those on the high-iron diet exhibited significantly elevated SI (Figure S2A), TIBC (Figure S2C), TF% (Figure S2D), as well as increased liver iron content (Figure S2E) and heart iron content (Figure S2F). These results indicated that the 2-week high-iron diet successfully induced an iron overload model in mice.

**Figure 4:**
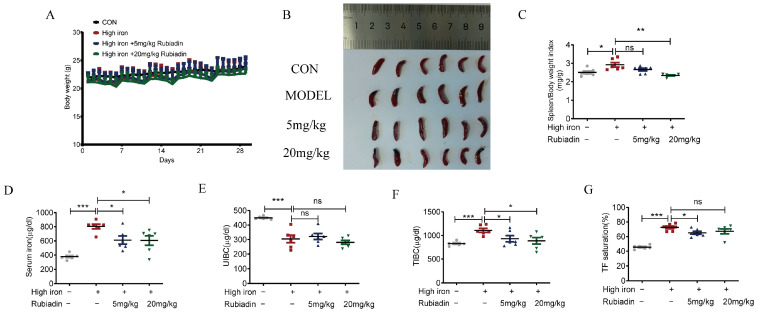
Rubiadin remarkably reverses the abnormal elevation of serum iron (SI) and alleviates splenomegaly caused by iron overload. Six-week-old mice with a successful high-iron-diet-induced iron overload model were continuously fed the high-iron diet for 4 weeks and intraperitoneally injected with different doses of rubiadin (5 mg/kg, 20 mg/kg). (**A**) Daily weight changes were recorded. (**B**) The size of the spleen in wild-type mice, model group mice, and rubiadin-treated mice was compared. (**C**) Spleen weight index (mg/g) was calculated. (**D**) SI and (**E**) unsaturated iron-binding capacity (UIBC) were measured. (**F**) Total iron-binding capacity (TIBC) and (**G**) transferrin saturation (TF%) were calculated. Significance was calculated using the two-way ANOVA. ns, no significance, * *p* < 0.05, ** *p* < 0.01, *** *p* < 0.001.

After the successful construction of the high-iron mice model, the mice were divided into high-iron model group, low dose (5 mg/kg) rubiadin group and high dose (20 mg/kg) rubiadin group. Rubiadin was administered daily for a continuous period of 4 weeks. Blood was taken from the heart after anesthesia, mice liver, heart, spleen, kidney, and duodenum were isolated. The results indicated that intraperitoneal injection of rubiadin did not significantly affect the body weight of the mice. (Figure 4A). Rubiadin significantly reversed splenomegaly (Figure 4B,C) and abnormal elevation of serum iron, TIBC, and TF% caused by high-iron feed (Figure 4D,F,G). There was no significant difference in UIBC among these groups (Figure 4E). Taken together, rubiadin significantly alleviated the splenomegaly, and reduced the serum iron and transferrin saturation caused by iron overload.

### 2.5. Rubiadin Remarkably Reverses the Elevation of Duodenal Iron Content and Further Enhances the Hepcidin mRNA and the Phosphorylation of STAT3 and SMAD1/5/9 Caused by Iron Overload

**Figure 5:**
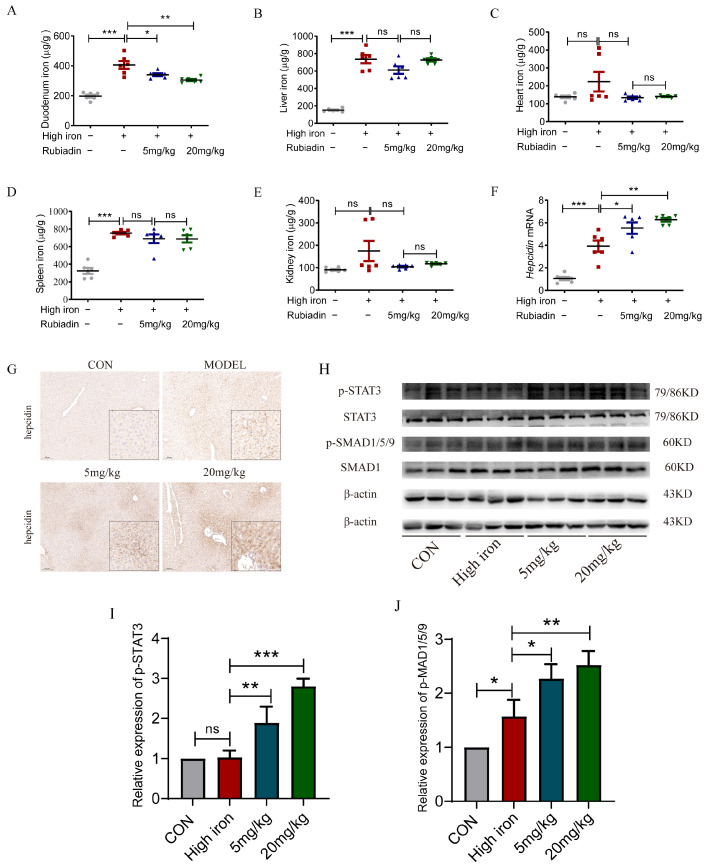
Rubiadin remarkably reverses the elevation of duodenal iron content and further enhances the hepcidin mRNA and the phosphorylation of STAT3 and SMAD1/5/9 caused by iron overload. The tissue samples were isolated and tested for iron in each organ. (**A**) Duodenal iron content. (**B**) Liver iron content. (**C**) Heart iron content. (**D**) Spleen iron content. (**E**) Kidney iron content. (**F**) *Hepcidin* mRNA levels in the liver of high-iron-fed mice after rubiadin treatment were measured by RT-qPCR. (**G**) Liver sections from different groups were immunohistochemically stained for hepcidin. Scale bars are 100 μm. (**H**) Western blotting assessed the phosphorylation levels of STAT3 and SMAD1/5/9 in the liver of the treated mice. Quantification of p-STAT3 (**I**) and p-SMAD1/5/9 (**J**) were shown. Significance was calculated using a two-way ANOVA. ns, no significance, * *p* < 0.05, ** *p* < 0.01, *** *p* < 0.001.

Intraperitoneal injection of rubiadin can significantly reverse the elevation of duodenal iron content caused by a high-iron diet (Figure 5A); there were no significant difference among other tissues (Figure 5B–E). Furthermore, the hepcidin mRNA level, protein level, and SMAD1/5/9 phosphorylation were augmented by high iron, while rubiadin further enhanced the expression of the hepatic hepcidin mRNA level, protein level, and the phosphorylation of SMAD1/5/9 based on high-iron feeding (Figure 5F–J), while the phosphorylation of STAT3 was not affected by the high-iron diet. Overall, decreased duodenal iron caused by rubiadin may be associated with its elevated hepatic hepcidin in high-iron-fed mice.

### 2.6. Rubiadin Upregulates Hepcidin via BMP6/SMAD1/5/9-Signaling Pathway

**Figure 6:**
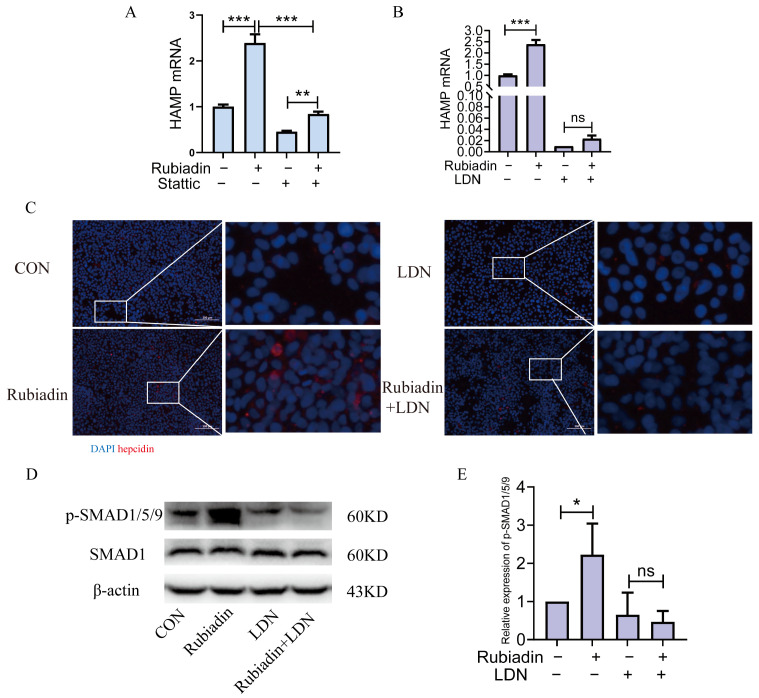
Rubiadin upregulates hepcidin via BMP6/SMAD1/5/9 signaling pathway. (**A**,**B**) Hepcidin mRNA was assessed in HepG2 cells treated with 20 μM rubiadin for 24 h, either with or without stattic or LDN-193189. (**C**) Hepcidin protein expression in HepG2 cells treated for 20 μM rubiadin in the presence or absence of LDN-193189 for 24 h was evaluated via an immunofluorescence assay. Scale bars are 100 μm. (**D**) Western blot analysis of phosphorylated SMAD1/5/9 in HepG2 cells treated with 20 μM rubiadin for 3 h in the presence or absence of LDN-193189 for 24 h. (**E**) Quantification of p-SMAD1/5/9 was shown. Significance was calculated using a two-way ANOVA. ns, no significance, * *p* < 0.05, ** *p* < 0.01, *** *p* < 0.001.

The above results suggest that rubiadin may regulate hepcidin through the IL-6/STAT3 and BMP6/SMAD1/5/9-signaling pathways, thereby alleviating iron overload in mice. Next, we performed in vitro validation. We used the STAT3 inhibitor stattic or the BMP6 inhibitor LDN-193189 co-cultured with rubiadin in HepG2 cells for 24 h. The results indicated that rubiadin could still increase the expression of hepcidin mRNA on the basis of using stattic (Figure 6A), while LDN-193189 could completely reverse rubiadin-induced upregulation of hepcidin (Figure 6B); cellular immunofluorescence also confirmed the same results (Figure 6C). Then, LDN-193189 was used to co-culture with rubiadin in HepG2 cells. As shown by the Western blot analysis, LDN-193189 was able to completely reverse the rubiadin-induced increase in SMAD1/5/9 phosphorylation. (Figure 6D,E). Collectively, these findings clearly indicated that rubiadin upregulated hepcidin expression through the BMP6/SMAD1/5/9-signaling pathway.

## 3. Discussion

This study highlights the critical role of rubiadin in modulating iron metabolism by up-regulating the expression of hepcidin. Our findings underscore the essential contribution of rubiadin to relieving iron overload. Furthermore, rubiadin reversed splenomegaly and elevation of serum iron caused by high-iron diet; notably, rubiadin further increased the hepcidin expression and the phosphorylation of SMAD1/5/9 on the basis of high-iron-fed mice. In addition, Rubiadin reversed the elevation of FtL induced by FAC. Our mechanistic investigations revealed that rubiadin upregulated hepcidin expression via a BMP6/SMAD1/5/9-dependent pathway.

Phlebotomy and iron chelation are the primary therapeutic approaches for managing iron overload. Nevertheless, these approaches have notable limitations, for example, frequent phlebotomy can result in anemia [15], while long-term use of iron chelating agents may lead to complications such as infections, gastrointestinal disorders, and skin damage [16]. As the key hormone of iron homeostasis, hepcidin has emerged as a crucial therapeutic target for managing iron-related disorders such as anemia and iron overload [17,18]. The ability of hepcidin analogs, such as mini-hepcidins, to alleviate iron overload has been investigated for hepcidin knockout mice [19]. Our study found that rubiadin significantly increased the expression of hepcidin at very low concentrations. We confirmed that rubiadin mitigated iron overload phenotype using high-iron-fed mice and FAC-treated HepG2 cells. The studies have shown that rubiadin could act as a potential compound to activate hepcidin.

Mutations in the HFE gene are the most common cause of hemochromatosis in adults, primarily characterized by iron overload in various organs and tissues, which results in insufficient hepcidin expression [20]. We successfully constructed a mice model of iron overload with a high-iron diet for 2 weeks, which was consistent with previous studies [21]. Our high-iron-diet-induced iron overload mice model closely imitated the pathological characteristics of hemochromatosis. Unlike the hereditary hemochromatosis mice model, the expression of hepcidin was increased in high-iron-fed mice, while rubiadin further increased the expression of hepcidin. In HFE, gene mutation induced hemochromatosis, and iron was accumulated in various organs, with the liver being the primary site of deposition. The excessive accumulation of iron in the liver leads to liver damage, which ultimately progresses to cirrhosis [22]. Interestingly, rubiadin significantly reduced duodenal iron content with no significant effect on liver iron; the probable reason was that rubiadin was not administered for a long enough time.

Previous studies have identified that the signaling pathways regulating hepcidin expression include the BMP6/SMAD-signaling pathway [9] and the IL-6-induced JAK2/STAT3-signaling pathway [10,11]. Genistein, a small molecule, has been shown to upregulate hepcidin expression in HepG2 cells via both STAT3-dependent and SMAD4-dependent pathways [23]. Previous studies have shown that adenine alleviates iron overload in mice by inducing hepatic hepcidin through the BMP6/SMAD1/5/9-signaling pathway [21]. Our results showed that the proteins of BMP6 and IL-6 were significantly induced by rubiadin. Moreover, rubiadin significantly enhanced the phosphorylation of STAT3 and SMAD1/5/9, while only the inhibitor of BMP6 could completely reverse the elevation of hepcidin induced by rubiadin.

## 4. Materials and Methods

### 4.1. Materials

Rubiadin was obtained using total synthesis according to the reported methods [24], followed by purification using silica gel column chromatography with a mixture of petroleum ether/ethyl acetate (10:1–5:1, *v*/*v*) as eluent. The purity of rubiadin was determined to be 99.8% by checking with HPLC-DAD-MS at 254 nm. Dimethyl sulfoxide was used to dissolve and preserve the rubiadin. Anti-mouse TfR1(Invitrogen, Cat. 13-6800, Waltham, MA, USA), anti-BMP6 (Invitrogen, Cat. MA5-35530). Anti-DMT1 (Cat. 20507-1-AP) and anti-mouse Fpn1 (Cat. 26601-1-AP) were purchased from Protein-tech company (Rosemont, IL, USA). Anti-FtL (Cat. ab69090), anti-FtH (Cat. ab65080), and anti-hepcidin (Cat. ab30760) were purchased from Abcam company (Waltham, MA, USA). Anti-p-STAT3 (Cat. 9145) and anti-p-SMAD1/5/9 (Cat. 13820) were purchased from CST company. Anti-STAT3 (Cat. A19566), anti-SMAD1 (Cat. A19113), and anti β-actin (Cat. AC028) were purchased from ABclonal company (Woburn, MA, USA). Anti-rabbit IgG, HRP-linked antibody (CST, Cat. 7074, Danvers, MA, USA), and anti-mouse IgG, HRP-linked antibody (CST, Cat. 7076). TRIzol reagent and PrimeScript RT Master Mix were obtained from Takara Bio Inc. MonAmpTM ChemoHS qPCR Mix (Monad Biotech, Shanghai, China), Stattic (MCE, Cat. HY-13818, Monmouth Junction, NJ, USA), LDN-193189 (Selleck, Cat. S2918, Houston, TX, USA).

### 4.2. Cell Culture

The human HepG2 cell line (ATCC) was cultured in Dulbecco’s Modified Eagle’s Medium (DMEM) (HyClone), supplemented with 10% fetal bovine serum (FBS) (Biowest), and maintained at 37 °C in a humidified atmosphere containing 5% CO_2_. HepG2 cells were trypsinized and seeded into 6-well plates at a density of 8 × 10^5^ cells per well. One day after plating, the cells were washed and treated with 100 μM ferric ammonium citrate (FAC) in the culture medium for 24 h [25]. Following medium removal, the cells were washed again and subsequently treated with 20 μM rubiadin for another 24 h. Western blot analysis was performed to evaluate the expression of transferrin receptor 1 (TfR1), divalent metal transporter 1 (DMT1), Fpn1, ferritin heavy chain (FtH), and ferritin light chain (FtL).

### 4.3. Animals

Six-week-old C57/BL6 male mice (Shanghai Jihui Laboratory Animal Center, Shanghai, China) were housed under SPF conditions. The mice were randomized to receive AIN-76A standard mouse diet (control group) or modified AIN-76A rodent diet with egg whites and 8.24 gm added carbonyl iron/kg (high-iron group) for 2 weeks to induce iron overload. Tissue and blood samples were collected to confirm successful model establishment. After model verification, the mice were treated with the respective doses daily for 4 weeks. CON I.P. group (standard diet, intraperitoneal injection solvent), MODEL I.P. group (high-iron diet, intraperitoneal injection solvent), LOW I.P. group (high-iron diet, 5 mg/kg rubiadin, intraperitoneal injection), HIGH I.P. group (high-iron diet, 20 mg/kg rubiadin, intraperitoneal injection. At the end of the treatment, blood was collected via cardiac puncture under anesthesia. The liver, heart, spleen, kidney, and duodenum were harvested and subjected to subsequent analysis. All studies were performed in accordance with the ARRIVE (Animal Research: Reporting of in vivo Experiments) guidelines and the Guide for the Care and Use of Laboratory Animals, as adopted and promulgated by the U.S. National Institutes of Health (NIH Publication No. 8023, revised 1996). The study protocol was reviewed and approved by the Institutional Animal Care and Use Committee at Shanghai University of Traditional Chinese Medicine (PZSHUTCM2307300008).

### 4.4. Cell Proliferation Detection

For the CCK-8 assay, cells (1 × 10^4^ well) were seeded in 96-well plates and cultured at 37 °C with 5% CO_2_. After incubation, 10 µL of CCK-8 reagent (YEASEN, Cat. 40203ES80, Shanghai, China) was added to each well. The absorbance was measured at 450 nm after 2 h.

### 4.5. Quantitative Real-Time PCR 

TRIzol Reagent (Takara Bio, Beijing, China) or EZ-Press RNA Purification Kit (EZBioscience, Roseville, MN, USA) Was Used to Extract Total RNA from Liver Tissue and Cells. Reverse Transcription Was Carried out Using PrimeScript RT Master Mix (TAKARA), and qPCR Was Performed Using MonAmp™ ChemoHS qPCR Mix (Monad Biotech) Following the Manufacturer’s Instructions. qPCR Was Conducted in Triplicate Using the QuantStudio 3 Real-Time PCR System (Applied Biosystems, Waltham, MA, USA), and at Least Three Independent Experiments Were Repeated.

Primers:

Human β-actin, forward: GAGCACAGAGCCTCGCCTTT,

reverse: ATCCTTCTGACCCATGCCCA;

Mouse β-actin, forward: AAATCGTGCGTGACATCAAAGA,

reverse: GCCATCTCCTGCTCGAAGTC;

Human hepcidin, forward: CAGCTGGATGCCCATGTTC,

reverse: CGCAGCAGAAAATGCAGATG;

Mouse hepcidin, forward: GCACCACCTATCTCCATCAACA,

reverse: TTCTTCCCCGTGCAAAGG [26].

Human IL-6, forward: ACTCACCTCTTCAGAACGAATTG,

reverse: CCATCTTTGGAAGGTTCAGGTTG;

Human BMP6, forward: AGCGACACCACAAAGAGTTCA,

reverse: GCTGATGCTCCTGTAAGACTTGA.

The Ct value of each target gene was standardized to that of β-actin mRNA, and the relative gene expression was determined using a 2^−ΔΔCt^ method.

### 4.6. Western Blot Analysis

The protein samples were collected and processed according to the method described previously [24]. Following centrifugation at 12,000 rpm for 15 min at 4 °C, the protein concentration was quantified using a BCA Protein Assay Kit (Yeasen, Shanghai, China). SDS-PAGE electrophoresis was performed to separate proteins based on their molecular weights. After electrophoresis, the proteins were transferred onto a PVDF membrane. The membrane was then blocked with 5% BSA or 5% skim milk powder for 2 h, followed by overnight incubation at 4 °C: anti-TfR1 (1:500), anti-DMT1 (1:1000), anti-Fpn1 (1:1000), anti-FtH (1:1000), anti-FtL (1:1000), anti-β-actin (1:3000), anti-STAT3 (1:2000) and anti-p-STAT3 (1:1000), anti-SMAD1 (1:1000), anti-p-SMAD1/5/9 (1:1000), and anti-BMP6 (1:1000). After an overnight washed with TBST three times for 10 min each, secondary antibodies (1:3000) were incubated for two hours at room temperature. The Immobilon Western Chemiluminescent HRP Substrate (Millipore, Darmstadt, Germany) detected the bands, and then they were quantified using ImageJ software, version 1.53q. A chemiluminescent detection system was used to automatically visualize the bands (Tanon Chemi Dog Ultra, Shanghai, China).

### 4.7. Cellular Immunofluorescence

HepG2 cells were seeded onto coverslips in 6-well plates to make cell slides. After treatment, the cells were washed twice with PBS, fixed with 4% paraformaldehyde for 30 min at room temperature, and subsequently washed three times with PBS. After washing, the fixed cells were then permeabilized with 0.2% Triton X-100 for 10 min followed by additional washing and then blocked with 3% BSA for 30 min at room temperature. Gently remove the blocking solution, dilute the anti-hepcidin antibody with PBS at a 1:200 ratio, and incubate the cell culture plate flat in a humidified chamber at 4 °C overnight. The next day, cells were incubated with appropriate secondary antibodies for 50 min at room temperature after being washed three times in PBS, each wash lasting 5 min. The coverslips were washed three times for 5 min each on a destaining shaker, which was placed in PBS (PH 7.4). After gently drying the sections, a DAPI stain was added dropwise to the circular area and incubated for 10 min at room temperature in the dark. The coverslips were washed three times with PBS (PH 7.4) for 5 min each time on a destaining shaker. After the slides are slightly spun dry, they are mounted with an anti-fluorescence quenching agent. Coverslips were observed using a fluorescence microscope (Nikon Corporation, NIKON ECLIPSE C1, Tokyo, Japan) and scanned using a scanner (3DHISTECH, Pannoramic MIDI, Budapest, Hungary).

### 4.8. Serum Iron and Transferrin Saturation

SI and unsaturated iron-binding capacity (UIBC) were measured according to the manufacturer’s instructions [27]. Total iron-binding capacity (TIBC) was calculated using the formula: TIBC (TIBC (μg/dL) = SI + UIBC), and transferrin saturation (Tf %) was calculated as follows: Tf % = SI/TIBC × 100.

### 4.9. Tissue Iron Measurement

A graphite furnace atomic absorption spectrophotometer was used to determine the total iron in the tissues, as described [28]. Briefly, tissues were first homogenized in 20 mM 4-(2-hydroxyethyl)-1-piperazine ethanesulfonic acid, then digested with an equal volume of ultrapure nitric acid. The samples were then analyzed using a graphite furnace atomic absorption spectrophotometer (Perkin-Elmer; Analyst 100, Waltham, MA, USA).

### 4.10. Immunohistochemistry

Mouse liver tissues were collected for paraffin embedding and sectioning. The section was pre-treated using heat-mediated antigen retrieval with sodium citrate buffer (pH 6, epitope retrieval solution 1) for 20 min. The section was then incubated with ab30760, 5 µg/mL, for 15 min at room temperature and detected using an HRP-conjugated compact polymer system. DAB was used as the chromogen. The section was then counterstained with hematoxylin and mounted with DPX. The sections were observed under a Nexcope microscope.

### 4.11. Enzyme-Linked Immunosorbent Assay (ELISA) Measurements

After 24 h of rubiadin treatment in HepG2 cells, IL-6 levels in cell culture supernatants were assessed using a Human IL-6 ELISA Kit (RayBiotech, Cat. ELH-IL6-1, Corners, GA, USA), according to the manufacturer’s protocol.

### 4.12. Statistical Analysis

Results were expressed as the mean ± standard error. Statistical significance was determined using a Student’s two-tailed *t*-test, one-way ANOVA, or two-way ANOVA with a Tukey’s post hoc analysis. Differences at *p* < 0.05 were considered to be statistically significant. Statistical significance indicated by asterisks: * *p* < 0.05, ** *p* < 0.01, *** *p* < 0.001. Statistical analysis was performed using GraphPad Prism software, version 8 (GraphPad Software, Inc., La Jolla, CA, USA).

## 5. Conclusions

In summary, this study demonstrated that rubiadin, a natural product extracted from the roots of rubiaceae plants, which can be obtained in large quantities by total synthesis, significantly upregulated the expression of hepcidin and decreased the expression of TfR1, Fpn1, and FtL. Our in vitro and in vivo studies have fully demonstrated that rubiadin mediated the upregulation of hepatic hepcidin alleviated cellular and mouse iron overload. Mechanically, rubiadin increased the expression of hepcidin in a BMP6/SMAD1/5/9-dependent manner. These results implied that using natural compounds, especially those derived from traditional Chinese medicine, to treat iron-overload-related disorders like hemochromatosis represents a promising strategy.

## Data Availability

Any additional information required to reanalyze the data reported in this paper is available from the lead contact upon request.

## Supplementary Materials

The following supporting information can be downloaded at: https://www.mdpi.com/article/10.3390/ijms26031385/s1.
