# Supplementary material for: Rubiadin Mediates the Upregulation of Hepatic Hepcidin and Alleviates Iron Overload via BMP6/SMAD1/5/9-Signaling Pathway
Source: Int J Mol Sci. 2025 Feb 6;26(3):1385. doi: 10.3390/ijms26031385 (PMC11818739; doi:10.3390/ijms26031385)
Supplement: Supplementary file 1 [file ijms-26-01385-s001.zip › ijms-3429816-supplementary.pdf]

Supplemental figures and Supplemental figure legends

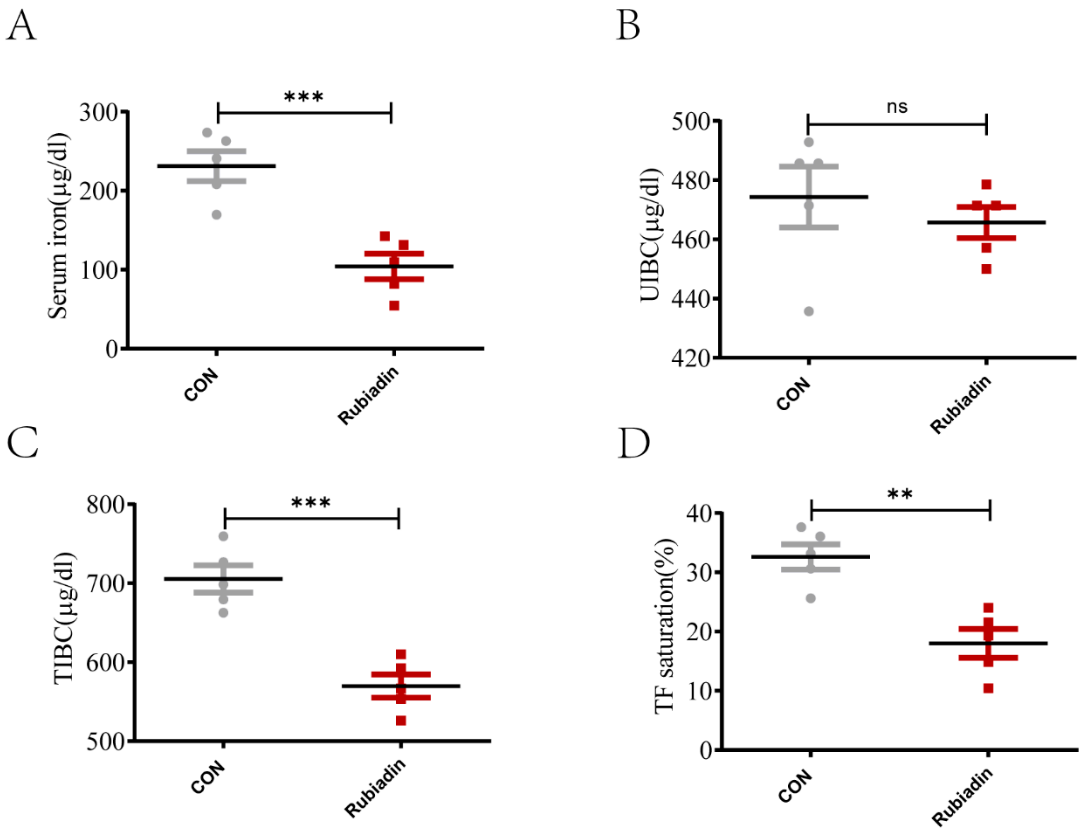

Supplemental Figure 1 Rubiadin significantly reduced serum iron, total iron-binding capacity, and transferrin saturation in C57BL/6 mice

(A) Serum iron, (B) UIBC, (C) TIBC, (D) TF saturation

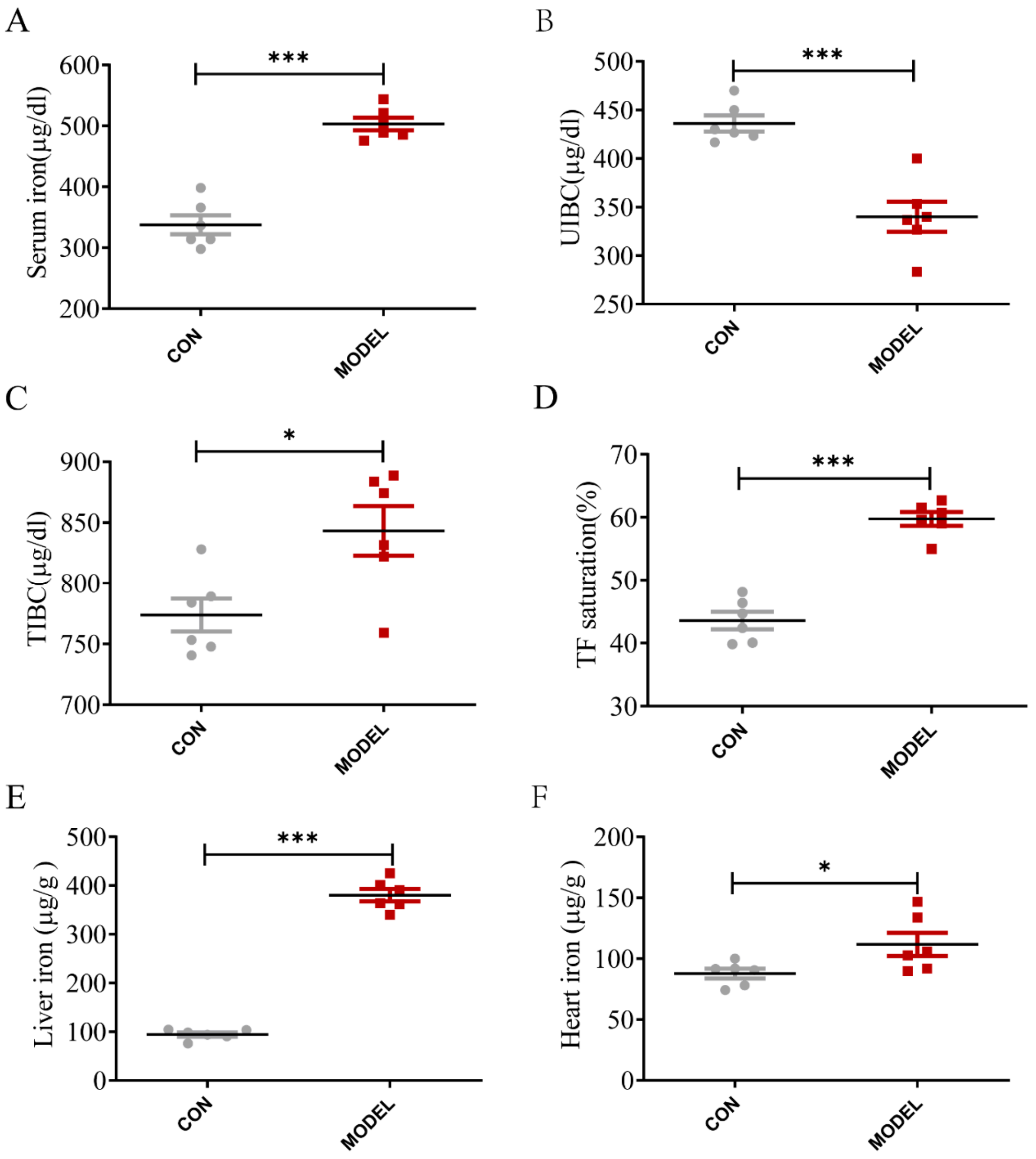

Supplemental Figure 2 Iron overload mice model were successfully constructed using high-iron diet

Mice were treated as described in "Materials and methods". The tissue samples were isolated and tested for iron content in each organ. (A) Serum iron, (B) UIBC, (C) TIBC, (D) TF saturation, (E) Liver iron content, (F) Heart iron content
